# Establishment and Verification of Prognostic Nomograms for Patients with Gastrointestinal Stromal Tumors: A SEER-Based Study

**DOI:** 10.1155/2019/8293261

**Published:** 2019-03-27

**Authors:** Zhan Chen, Rui-Min Lin, Yue-Kui Bai, Yue Zhang

**Affiliations:** ^1^Department of General Surgery, Beijing Haidian Hospital, Beijing Haidian Section of Peking University Third Hospital, Beijing, China; ^2^Department of Hematology, Beijing Luhe Hospital, Capital Medical University, Beijing, China

## Abstract

With gastrointestinal tract as the origin, gastrointestinal stromal tumor (GIST) is recognized as the very widespread mesenchymal tumor. A precise prognostic model of survival is required to guide the treatment options of patients with GIST. This study was designed to map the overall survival (OS) and cancer-specific survival (CSS) of GIST patients. According to the Surveillance, Epidemiology, and End Results (SEER) program database, we acquired the data of 6,713 patients with GIST who were diagnosed between 2004 and 2014. We randomly separated the patients into training (n = 4,699) and validation (n = 2,014) groups. To assess the prognostic impact of multiple clinical parameters, the Kaplan-Meier approach and the Cox proportional hazards regression model were adopted, where essential prognostic variables were combined to create nomograms. The consistency index and curve of calibration had been adopted to assess nomogram discrimination ability and prediction accuracy. A multifactor analysis of the training cohort showed that age, gender, size of tumor, location, and primary surgery were remarkably related to survival, and these variables were applied to create nomograms. The nomogram demonstrated excellent accuracy in estimating 2-, 3-, and 5-year OS and CSS, with a C-index of 0.740 (95% confidence interval [CI], 0.723-0.757) for OS and 0.743 (95% CI, 0.718-0.768) for CSS. In the validation cohort, the nomogram-predicted C-index was 0.741 for OS (95%CI, 0.717-0.765) and 0.746 (95%CI, 0.713-0.779) for CSS. All calibration curves showed good consistency between predicted and actual survival. A new nomogram was created and verified to predict the OS and CSS of patients with GIST. These new prognostic models can help enhance the accuracy of survival outcome predictions, thus facilitating to provide constructive therapeutic suggestions.

## 1. Introduction

With the gastrointestinal tract as the cause, gastrointestinal stromal tumor (GIST) is seen as the very widespread mesenchymal tumor, and most patients with GIST exhibit activation of the KIT and platelet-derived growth factor receptor (PDGFRA) mutations [1, 2]. Surgical resection is the main management tool applied by local attending physicians; however, tumor recurrence is familiar in patients with GIST [3]. After the use of tyrosine kinase inhibitors, the treatment of metastatic GIST has changed [4, 5]. Nevertheless, the determination of prognostic variables and the creation of staging systems and precise prognostic models based on large sample studies are restricted by the rareness of these tumors.

Due to the rareness of GIST, large databases like the Surveillance, Epidemiology, and End Results database (SEER database) can be used to be an excellent database to explore these tumors. The SEER database is kind of a population-based cancer registration system of the USA taking 28% Americans into account. The database can function as a resource for examining familiar cancer incidence and result patterns. Thanks to the wide range of data on cancer, the SEER database is a distinctive resource to study special malignancies. Nomograms are graphical representations of mathematical models, where information about certain features is used together to estimate specific endpoints. The handy graphical display of the nomogram makes it possible to make easy and rapid predictions in clinical practice [6]. By integrating different essential factors, the nomogram can output individual predictions of the possibility of events, like the individual possibility of death or illness recurrence [7]. Thus, nomograms dependably predict the clinical outcomes of a great number of types of cancer [8–10].

In this research, to determine the risky variables related to the overall survival (OS) and cancer-specific survival (CSS) of patients with GIST, patient records from the SEER Cancer Registry were acquired and used to establish nomogram for the prediction of the prognoses of patients with GIST.

## 2. Material and Methods

### 2.1. Data Source

Data extracted from the SEER registry database of the National Cancer Institute were used in this research. The SEER database gathers information related to cancer prevalence, incidence, factors based on population, mortality, major tumor features, and management at 18 registries in the USA.

### 2.2. Study Population

We used SEER *∗* Stat software (Version 8.3.2) to analyze the data from patients who were diagnosed between 2004 and 2014, which represented the patients with GIST patients who were studied in this analysis. For each patient, the following data was acquired: year of diagnosis, age at diagnosis, gender, race, size of tumor, location, surgery of primary, information of survival, and reason lead to death. A total of 6713 patients with GIST were inconsistently separated into two groups (training group, n = 4699 and validation group, n = 2014). Patients in the SEER database who were classified as American Indians / Alaska Aboriginal or Asian / Pacific Islanders were categorized into the “others” race category to conduct the analysis.

### 2.3. Creation of the Nomograms

The training group was used to establish a line diagram. One of the main endpoints of interest was OS, which was identified as the time from diagnosis to death from any cause. In the OS analysis, patients who remained alive at the last follow-up were considered as censored observations. The other major endpoint of interest was CSS, which was defined as the time from diagnosis to death due to GIST. In the CSS analysis, patients who died of other causes or remained alive at the last follow-up were classified with respect to their last examination.

Kaplan-Meier and Cox proportional hazards regression models were adopted to identify survival-associated variables. Significant variables that associated with survival were investigated in univariate or multivariate analysis (*p* < 0.05) and applied to construct nomograms of OS and CSS.

### 2.4. Nomogram Confirmation

To internally validate the training cohort and externally validate the validation cohort, 1000 bootstrap resamples of nomograms were conducted. The calibration curves were created using the marginal estimate and the model average prediction probability. In a perfect calibration model, the forecast should fall on the calibration curve as a 45° diagonal line. Using the C-index to evaluate the predictive performance had similarity with the area beneath the curve (AUC) calculations, but seems kind of proper for censored data [11]. Larger C-indices represent more precise prognostic estimates [12].

### 2.5. Statistical Analysis

To carry out statistical analyses, IBM SPSS statistics 22 software (SPSS Inc., Chicago, IL, USA) was used. The Kaplan-Meier method and Cox proportional hazards regression model were adopted to identify survival-related variables. We investigated the variables that remarkably correlated with survival in univariate or multivariate analyses.

With the multivariate analysis results as the basis, by means of R 3.4.1 software (Institute for Statistics and Mathematics, Vienna, Austria; http://www.r-project.org/), a nomogram was constructed. We used “RMS” R library (cran.r-project.org/web/packages/rms) to develop survival models. The bilateral *p* values <0.05 reveal that it owns the statistical significance.

## 3. Results

### 3.1. Clinicopathologic Features of Patients

In general, 6713 patients with GIST were defined in the SEER database. Patients were inconsistently separated into training (n = 4699) and validation (n = 2014) groups.  Table 1 summarizes the clinicopathological features from the SEER database of the training and validation cohorts. There were no substantial differences between the two groups.

### 3.2. OS and CSS of Training Cohort

The median follow-up times were 45 months (1 to 131 months), 2 years, 3 years, and 5 years, with OS rates of 84.4%, 79.4%, and 70.1%. The 2-, 3-, and 5-year CSS rates were 92.4%, 89.2%, and 83.6%.

### 3.3. Independent Prognostic Variables in the Training Group

**Figure 1:**
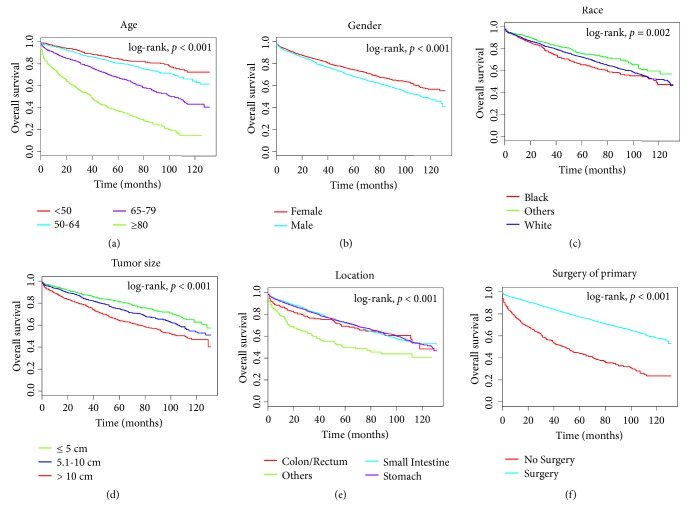
Kaplan-Meier survival curves for overall survival in the training cohort, as stratified by (a) age, (b) gender, (c) race, (d) tumor size, (e) location, and (f) surgery of the primary tumor. The race of others includes American Indian/Alaskan Native, and Asian/Pacific Islander; locations of others include esophagus, appendix, and peritoneum.

**Figure 2:**
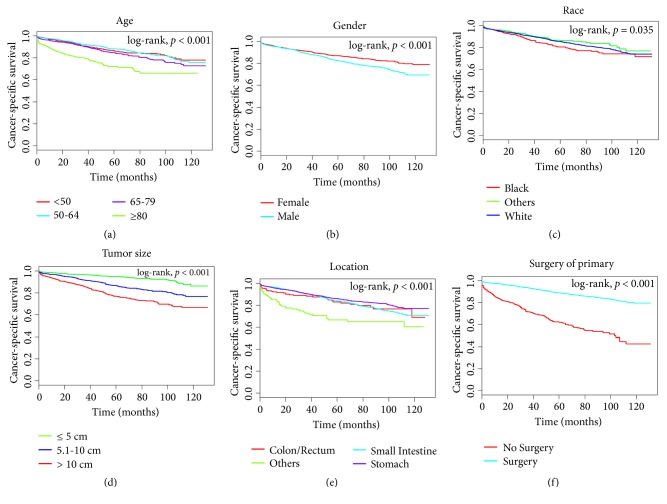
Kaplan-Meier survival curves for cancer-specific survival in the training cohort, as stratified by (a) age, (b) gender, (c) race, (d) tumor size, (e) location, and (f) surgery of primary. The race of others includes American Indian/Alaskan Native and Asian/Pacific Islander; locations of others include esophagus, appendix, and peritoneum.

Univariate and multivariate analyses were conducted to identify predictors of OS and CSS among the 4699 patients in the training cohort. As can be seen in Figures 1 and 2, age at diagnosis, gender, race, size of tumor, position, and surgery of the primary tumor significantly associated with OS and CSS in univariate survival analyses with the Kaplan-Meier method. These factors were deeply compared using the log-rank test (*p* < 0.05). The Cox proportional hazards regression model was used to deeply study the impacts of different factors. The OS and CSS multivariate analyses identified higher hazard ratios (HRs) for the following features: older age, male gender, size of tumor > 10 cm, nongastric location, and no surgery of the primary tumor (*p* < 0.05) (Table 2).

### 3.4. Prognostic Nomograms for OS and CSS

**Figure 3:**
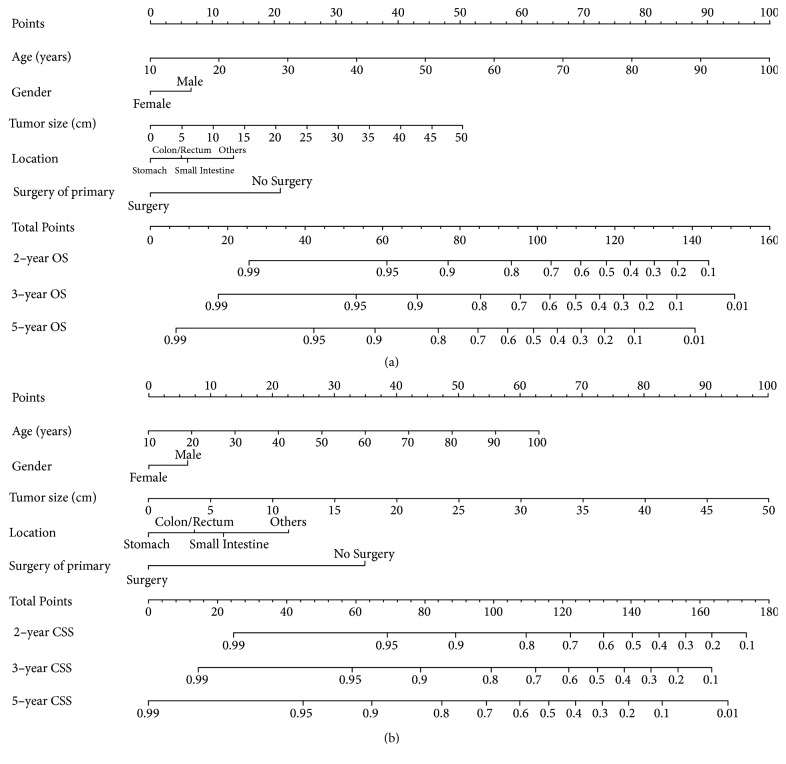
Nomograms for predicting the 2-, 3-, and 5-year (a) overall survival and (b) cancer-specific survival of GIST. Abbreviations: OS, overall survival; CSS, cancer-specific survival.

All remarkable independent factors of the Cox proportional hazards regression in the training group were included in the prognostic nomogram.  Figure 3(a) shows the OS nomogram at the second year, the third year, and the fifth year, and Figure 3(b) presents the CSS nomogram at second year, the third year, and the fifth year. By integrating the scores related to each variable and projecting the overall scores to the bottom scale, the possibility of OS and CSS at 2, 3, and 5 years can be predicted.

Generally, there were excellent OS and CSS rates for younger patients, female, smaller tumors, stomach positions, and patients receiving primary tumor surgery. With the help of the nomogram, prognoses can be effectively predicted based on personal patient features.

### 3.5. Confirmation of the Nomograms

**Figure 4:**
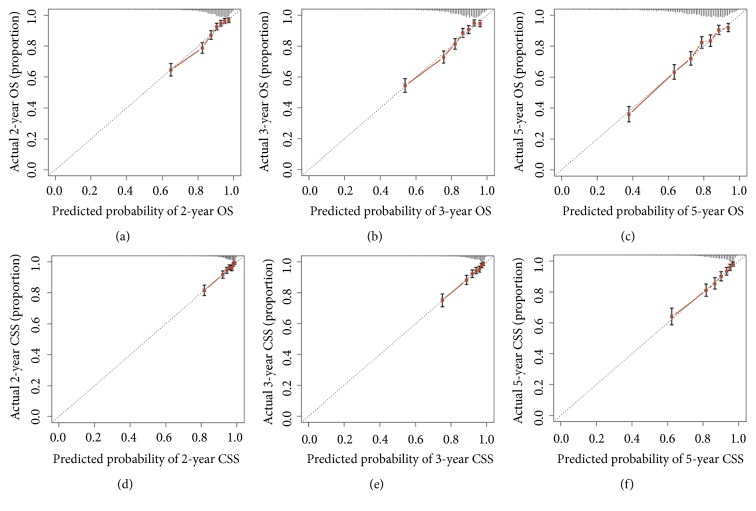
The calibration curves for predictions of overall survival (a-c) and cancer-specific survival (d-f) in the training cohort at 2, 3, and 5 years after diagnosis. The dashed line represents perfect agreement between the nomogram-predicted probability (x-axis) and the actual probability, calculated from a Kaplan-Meier analysis (y-axis). Abbreviations: OS, overall survival; CSS, cancer-specific survival.

**Figure 5:**
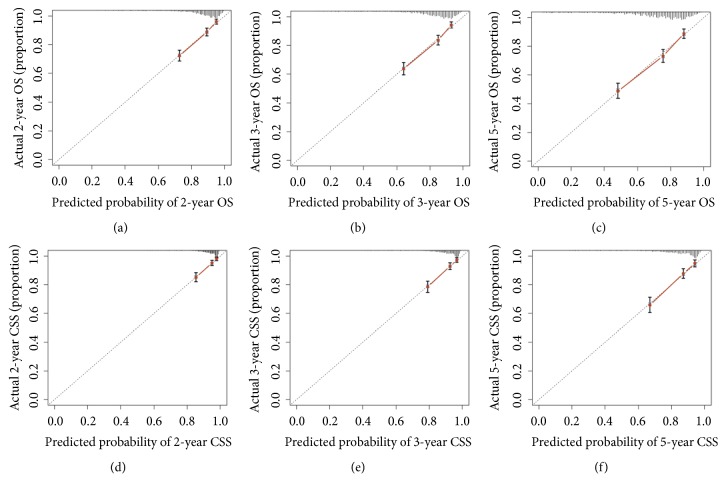
The calibration curves for predictions of overall survival (a-c) and cancer-specific survival (d-f) in the validation cohort at 2, 3, and 5 years after diagnosis. The dashed line represents perfect correspondence between the nomogram-predicted probability (x-axis) and the actual probability calculated from a Kaplan-Meier analysis (y-axis). Abbreviations: OS, overall survival; CSS, cancer-specific survival.

The verification of the nomogram was carried out by a 1000 resampling bootstrap analysis, in internal and external way. The analysis of the internal validation group (training group) demonstrated that the C-index of OS estimate was 0.740 (95% CI, 0.723-0.757), and the predictive value of CSS was 0.743 (95% CI, 0.718-0.768). Analogously, the C-index values to evaluate OS and CSS were 0.741 (95% CI, 0.717-0.765) and 0.746 (95% CI, 0.713-0.779) (Table 3), in the external validation cohort. These outcomes showed that the nomogram model was quite precise. The internal and external calibration curves in the training and validation groups (Figures 4 and 5) showed good consistency between the predicted and observed OS and CSS values for 2, 3, and 5 years.

## 4. Discussion

Accurate risk stratification of GIST is critical for treatment selection and prognosis assessment. Several classification systems to prognosticate GIST have been proposed. The two most widely accepted risk classification systems are the US National Institute of Health (NIH) criteria and the Armed Forces Institute of Pathology (AFIP) criteria. The NIH consensus classification system, based on tumor size and mitotic count, is commonly used to assess patient prognosis after surgical resection [13]. The AFIP has suggested a commonly adopted risk classification method that includes the major tumor sites, mitotic counts, and size of tumor [14, 15]. More recently, Joensuu et al. proposed a modified consensus criteria considering that in addition to tumor size and mitotic count, tumor location and tumor rupture appeared to be important prognostic factors for completely resected GISTs [16]. These classifications systems have been compared and validated by several investigators who have also proposed modifications, including additional factors such as tumor histologic subtype [17, 18], ulceration and mucosal invasion [19, 20] to be included within the risk factors. However, the optimal staging system based on large sample studies was not yet developed and examined.

The nomogram, a graphical display of a mathematical model, combines biological and clinical variables for the identification of the probability of clinical events. Compared with the existing tumor staging system, nomograms achieve more excellent prediction precision and prognostic value [21–23]. Based on the size of tumor, location, and mitotic index of 127 patients, from 1983 to 2002, the Memorial Sloan Kettering Cancer Center (MSKCC) developed a prognostic nomogram that predicted the risk for tumor recurrence after surgical resection of a localized primary GIST. This nomogram prediction for recurrence-free survival (RFS) might be more accurate than the predictions of the AFIP-Miettinen system, which can be adopted to choose patients who will benefit by Imatinib therapy [24]. Chok et al. validated the MSKCC prognostic nomogram of 289 patients GISTs and compared its predictive accuracy against other established risk classification systems, including the NIH, AFIP, and Joensuu criteria. The MSKCC nomogram slightly underestimated the probability of RFS after surgical resection of GISTs, although it had a significantly better predictive accuracy compared to the NIH and Joensuu prognostic indexes [25].

Since the SEER project includes data from a large number of hospitals and almost 30% of the overall population of the country, the contained data should allow the widespread application of nomogram in clinical practice decisions.

Taking the relatively long-term survival potential of GIST into consideration, the death of patients with GIST is often due to non-GIST causes. Therefore, the OS predictions may not accurately reflect the long-term survival of GIST. Thus, when predicting the CSS of GIST, other causes of death should be considered.

In this research, a nomogram was established with 6713 patients the basis from the SEER database and used to predict the 2-, 3-, and 5-year OS and CSS rates of patients with GIST based on five important factors: age at diagnosis, gender, size of tumor, location, and primary tumor surgery. The aim of this study was to effectively predict patient prognosis from concrete patient features. The nomogram's discrimination performance was assessed by an internal bootstrap resampling approach. The C-index showed the ability of nomogram to estimate OS and CSS rates of patients with GIST at 2, 3, and 5 years.

As demonstrated in the nomogram, age as well as gender was associated with OS and CSS, regardless of size of tumor and location. In previous studies, age was proven a significant predictor and prognostic variable [26, 27]. Our results show that OS and CSS rates were more considerable for female. Therefore, our nomogram prognosis model can increase prediction accuracy by adding two prognostic factors: age and gender.

GIST can take place along every part of the gastrointestinal tract and is highly widely related to the stomach (60-70%) and small intestine (25-30%). The contribution of colorectal GIST (5-10%) and esophageal GIST (1%) to the overall incidence is very small [14]. Recently, gastric GIST has been found to be more advantageous than GIST in the small intestine, so location is usually considered as prognostic variable [14, 28–30]. Our findings indicate that the prognosis of patients with gastric GIST is more excellent than that of those with GIST at other locations, and the disease location can serve as an absolute prognostic variable for survival.

There are several restrictions to this study. Firstly, as a retrospective study, it suffers from inherent and inevitable biases. Therefore, in order to confirm the results, large-scale, randomized, and controlled studies are needed. Second, the mandatory use of Imatinib or other new, targeted agents in the high-risk group of GIST patients after surgery modifies substantially the clinical outcome and the prognosis of OS and CSS. However, the SEER database offers no data on important treatments, like tyrosine kinase inhibitors. In addition, many other factors that may affect patient prognosis, such as histological grade, mitotic index, tumor rupture, and the existence of mutation of the KIT gene and exon mutation (exon 11 versus exon9, exon13, exon17) as well as with the PDGRA mutation (exon 12, exon14, exon18). Moreover, new mutations have been described that may influence the clinical outcome and the response to therapy with new drugs of GIST, such as BRAF mutation, SDH deficiency, and NF-1 mutant [31–35]. However, information on these factors is not available in the SEER Cancer Registry, so these potential prognostic factors were not included in the nomogram. Regardless of these restrictions, this study pioneers the use of a nomogram to estimate the survival rates of patients with GIST patients on a large population analysis.

Current studies demonstrate that age, gender, size of tumor, location, and primary tumor surgery are absolute risk variables for survival GIST patients. According to specific patient characteristics, the OS and CSS rates at 2, 3, and 5 years could be accurately predicted with the aid of nomograms, which can facilitate the ability of clinicians to determine extremely risky patients and perform more accurate survival assessments.

## 5. Conclusions

We created and examined a new nomogram that can evaluate OS and CSS in patients with GIST, and these brand-new prognostic methods can help improve survival predictions and facilitate reasonable treatment recommendations.

## Figures and Tables

**Table 1:** Characteristics of the training and validation cohorts.

Characteristic	All Patients (n = 6713)		Training Cohort (n = 4699)		Validation Cohort (n = 2014)	
	No.	%	No.	%	No.	%
Age at diagnosis, years						
Median	63.4±14.3		63.4±14.3		63.5±14.2	
Range	10 - 101		10 - 101		14 - 101	
Gender						
Male	3522	52.5	2442	52.0	1080	53.6
Female	3191	47.5	2257	48.0	934	46.4
Race						
White	4607	68.6	3266	69.5	1341	66.6
Black	1217	18.1	818	17.4	399	19.8
Others^a^	846	12.6	588	12.5	258	12.8
Unknown	43	0.7	27	0.6	16	0.8
Tumor size						
⩽ 5 cm	2459	36.6	1723	36.7	736	36.6
5.1-10 cm	2017	30.1	1424	30.3	593	29.4
> 10 cm	1503	22.4	1036	22.0	467	23.2
Unknown	734	10.9	516	11.0	218	10.8
Location						
Stomach	4241	63.2	2974	63.3	1267	62.9
Small intestine	1910	28.5	1313	27.9	597	29.6
Colon/Rectum	334	5.0	242	5.2	92	4.6
Others^b^	228	3.3	170	3.6	58	2.9
Surgery of primary						
No surgery of primary tumor	1228	18.3	855	18.2	373	18.5
Surgery of primary tumor	5420	80.7	3804	81.0	1616	80.3
Unknown	65	1.0	40	0.8	25	1.2

Others^a^ includes American Indian/Alaskan Native and Asian/Pacific Islander; Others^b^ includes esophagus, appendix, and peritoneum.

**Table 2:** Multivariate analysis of overall survival and cancer-specific survival in the training cohort.

Variable	OS			CSS		
	HR	95% CI	*P *	HR	95% CI	*P *
Age at diagnosis, years			0.020			0.040
<50	ref			ref		
50-64	1.393	1.143 to 1.781		1.477	1.034 to 1.735	
65-79	1.309	1.098 to 1.816		1.568	1.155 to 2.199	
*⩾*80	1.719	1.028 to 2.875		1.639	1.265 to 2.207	
Gender			0.000			0.008
Female	ref			ref		
Male	1.397	1.230 to 1.585		1.269	1.063 to 1.516	
Race			0.236			0.401
White	ref			ref		
Black	1.380	0.870 to 2.190		1.485	0.772 to 2.857	
Others^a^	1.155	0.922 to 1.447		1.153	0.836 to 1.589	
Tumor size			0.000			0.000
⩽ 5 cm	ref			ref		
5.1-10 cm	2.596	2.135 to 3.156		1.686	1.313 to 2.164	
> 10 cm	2.314	1.891 to 2.832		1.654	1.279 to 2.139	
Location			0.001			0.000
Stomach	ref			ref		
Small intestine	1.174	1.017 to 1.355		1.393	1.141 to 1.700	
Colon/Rectum	1.115	0.847 to 1.467		1.282	0.884 to 1.861	
Others^b^	1.679	1.293 to 2.179		1.920	1.348 to 2.734	
Surgery of primary			0.000			0.000
No surgery of primary tumor	ref			ref		
Surgery of primary tumor	0.327	0.286 to 0.375		0.242	0.201 to 0.292	

Others^a^ includes American Indian/Alaskan Native and Asian/Pacific Islander; Others^b^ includes esophagus, appendix, and peritoneum.

Abbreviations: OS, overall survival; CSS, cancer-specific survival; HR, hazard ratio; CI, confidence interval; ref, reference.

**Table 3:** The C-indices for nomogram predictions of overall survival and cancer-specific survival.

Group	OS		CSS	
	C-index	95% CI	C-index	95% CI
Training cohort	0.740	0.723 to 0.757	0.743	0.718 to 0.768
Validation cohort	0.741	0.717 to 0.765	0.746	0.713 to 0.779

Abbreviations: OS, overall survival; CSS, cancer-specific survival; C-index, index of concordance; CI, confidence interval.

## Data Availability

Relevant data can be accessed through proper request, from the first author.

## Abbreviations

AFIP: Armed Forces Institute of Pathology
AUC: Area under the curve
C-index: Index of concordance
CI: Confidence interval
CSS: Cancer-specific survival
GIST: Gastrointestinal stromal tumors
HR: Hazard ratio
MSKCC: Memorial Sloan Kettering Cancer Center
NIH: National Institute of Health
OS: Overall survival
PDGFRA: Platelet-derived growth factor receptor alpha
RFS: Recurrence-free survival
SEER: Surveillance, Epidemiology, and End Results.
